# A mechanistic investigation of low salinity water flooding coupled with ion tuning for enhanced oil recovery†

**DOI:** 10.1039/d0ra08301a

**Published:** 2020-11-24

**Authors:** Rohit Kumar Saw, Ajay Mandal

**Affiliations:** Chemical Flooding Laboratory, Department of Petroleum Engineering, Indian Institute of Technology (Indian School of Mines) Dhanbad 826004 India ajay@iitism.ac.in

## Abstract

Oil recovery by low salinity water flooding (LSWF) from carbonate reservoirs has gained tremendous attention in recent years due to its cost-effectiveness and environment-friendly nature. The mechanisms of low salinity water flooding for enhanced oil recovery are very complex and depend on the mineralogy of the formation rock, properties of injection brine and reservoir fluids. The present work aimed at the optimization of salinity and concentration of potential determining ions (PDIs) in injection water for enhanced oil recovery from carbonate reservoirs. Initially, we conducted a series of experiments on the dilution effect of seawater (SW) with the help of rock/fluid and fluid/fluid interactions *via* interfacial tension (IFT), zeta potential and contact angle measurements. This offered an optimum salinity (20dSW) with an 11% increase in recovery of the original oil in place (OOIP) over the SW injection in secondary flooding mode. Then, the ion tuning was done on the optimum salinity (20dSW) by manipulating the PDIs (Ca^2+^, SO_4_^2−^ and Mg^2+^) while keeping ionic strength constant. The properties of ion tuned brine were optimized by zeta potential and contact angle measurements. The core flooding experiments performed with the injection of designed ion tuned water obtained by dilution and ion tuning of SW showed more than 20% OOIP as incremental recovery over the SW injection. Effluent analysis after the flooding confirms that the main mechanisms for enhanced oil recovery include calcite dissolution and wettability alteration due to interplay of PDIs.

## Abstract 2

The combined effects of dilution and ion tuning of seawater for enhanced oil recovery from carbonate reservoirs. Dominating mechanisms are calcite dissolution and the interplay of potential determining ions that lead to wettability alteration of rock surface.

## Graphical abstract

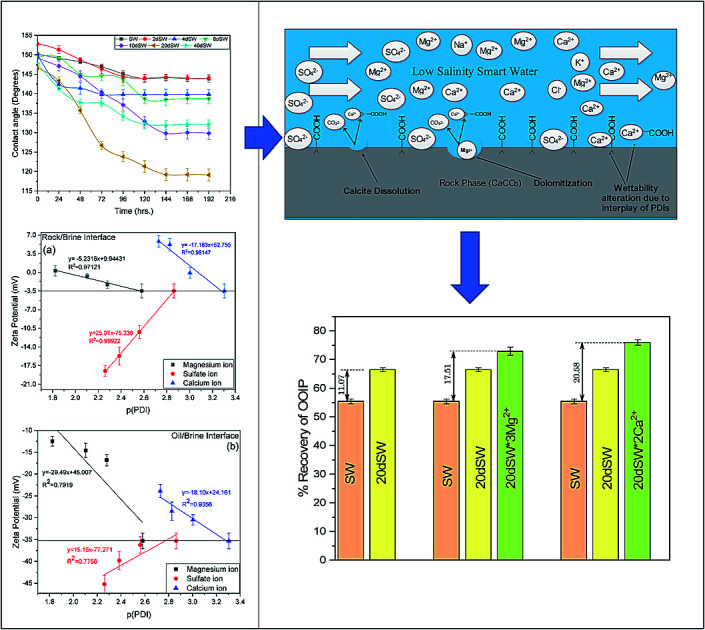

## 1. Introduction

Out of all the proven oil reserves found in the world, carbonate reservoirs contain more than half of it.^1^ Carbonate reservoirs are mainly composed of calcite and dolomite with impurities such as quartz, anhydrite, aragonite and clay minerals.^2^ After primary recovery, most of the oil remains trapped in the reservoirs due to complex wettability of porous media and rock–fluid interactions^3,4^ which lead to enormous motivation to develop the techniques which are both cost effective and environment friendly for recovering as much oil as possible. Low salinity water flooding (LSWF) has proven its ability as one of the most promising approaches in this regard. Numerous experimental works have been reported in the literature using different types of measurements like IFT, zeta potential, contact angle, spontaneous imbibition, core flooding *etc.* using sea water, low salinity water and smart water as injection fluids particularly in carbonate reservoirs. Though significant contributions have been made by different researchers, the mechanisms of LSWF in carbonate reservoir are still not well understood due to the complex nature of the carbonate reservoirs and the conflicting reported results.^5^

Past studies on LSWF in carbonate reservoir were mainly focused on two different approaches which are: (a) the successive dilution of formation brine/sea water and their injection into the reservoir in secondary and tertiary mode, thus altering the salinity and ionic strength of injection brine,^6–9^ and (b) the tuning the ionic composition of sea water keeping the ionic strength constant and by altering the concentration of SO_4_^2−^, Ca^2+^, Mg^2+^ also termed as potential determining ions (PDIs) for carbonate reservoirs.^10–14^ Both the approaches have proved their ability to increase oil recovery.

Yousef *et al.*^15^ have demonstrated the positive effect of LSWF on limestone reservoir cores with the dilution of formation water and sea water as an injection fluid. They showed that injection of different dilutions of sea water sequentially led to an increase in incremental oil recovery of 7–8.5% from 2 times diluted, 9–10% from 10 times diluted and 1–1.6% from 20 times diluted sea water flooding. Zaeri *et al.*^16^ studied the effect of temperature, salinity, permeability and connate water saturation by spontaneous imbibition studies for LWSF. They also reported that the recovery at optimum salinity increased with an increase in temperature. In another study, Nasralla *et al.*^17^ showed the effect of salinity and mineralogy of reservoir carbonate cores on enhanced oil recovery by both imbibition test and core flooding. It was also stated that oil recovery by LSWF could not be increased upon further dilution after the optimum salinity.

The effect of PDIs of sea water and temperature in altering the wettability of rock towards more water wet was first investigated by Austad and their co-workers.^12,18–21^ It was observed that sea water spiked with different PDIs concentrations at constant ionic strength could enhance the oil recovery to a greater extent. Performance of ion tuned water flooding in carbonate rocks is generally dependent on reservoir temperature,^22^ crude oil composition, rock mineralogy^6^ and water chemistry of injection and formation water.^23^ According to some recent studies, the efficiency of LSWF in carbonate reservoirs is dependent on the following conditions: (i) combination of PDIs with a lower concentration of monovalent ions (ii) presence of anhydrite and (iii) temperature should be above 70 °C.^24,25^ However, the meeting of all the above criteria doesn't ensure the positive effect of LSWF due to the heterogeneity and complex nature of different reservoirs.

Changing the ionic composition has shown a significant impact on oil recovery. Shehata *et al.*^26^ evaluated the brine salinity and effect of ions both in the secondary and tertiary mode of oil recoveries in limestone rocks at 90 °C, and concluded that Ca^2+^, SO_4_^2−^ and Mg^2+^ played an important role in oil mobilization. In another study, it was shown that tuning the ionic composition of injection brine can reveal the answer to why the LSWF effect is not always observed.^27^ It is also reported that tuning the injection brine can lead to more favorable results. Purswani and Karpyn^28^ conducted the flooding experiment to understand the effects of PDIs (Ca^2+^, SO_4_^2−^ and Mg^2+^) on carbonate rocks with sea water. They showed that all chemically tuned brines showed a higher recovery than the plain sea water with maximum recovery obtained with Mg^2+^ spiked brine than the Ca^2+^ and SO_4_^2−^ spiked brine due to higher activity of Mg^2+^ ion at the higher temperature. They also proposed that chemical reaction includes salt precipitation, crude oil desorption/solubilization, and mineral dissolution. Authors^19,29^ have also shown that the tuning of the composition of sea water have an optimum condition for maximum oil recovery. They stated that increasing the sulfate concentration beyond 4 times can lead to a detrimental effect on oil recovery. Fathi *et al.*^19^ reported this optimum condition in NaCl free sea water whereas Awolayo *et al.*^29^ described this optimum condition in sea water.

From the prior literature survey, it is clear that both the methods of dilution and ion tuning of injection brine have got their individual benefits in extracting the original oil in place (OOIP). Combining these two potential methods can lead to a more efficient enhanced oil recovery method. In this paper, an attempt has been made to combine both the dilution and ionic tuning of injection water to enhance the recovery of oil in carbonate reservoirs. Sea water and diluted sea water are used as an injection fluid in most of the carbonate reservoirs in the offshore region. The effect of dilution of sea water was evaluated by measurement of rock–fluid and fluid–fluid properties by IFT, zeta potential and contact angle along with core flooding. The assessment of the salinity effect on interfacial and physicochemical properties was done to determine an optimal salinity. The properties were further improved by tuning the PDIs concentration 2 times, 3 times and 4 times of each of PDIs. This type of water is commonly known as ion tuned water. Core flooding experiments were performed to evaluate the efficiency of the designed ion tuned water for enhanced oil recovery. The synergistic effects of both the techniques *i.e.*, salinity effect and ion tuning lead to the enhanced effect on oil recovery compared to that achieved individually.

## 2. Experimental setups and methodology

### 2.1 Crude oil

Crude oil contains thousands of different hydrocarbon molecules. Characterization of the individual molecule is not possible and hence group type characterization is generally employed for crude oil and Saturates–Aromatics–Resins–Asphaltene (SARA) analysis is one of them.^30^ Saturates and aromatics are classified as non-polar compounds whereas resin and asphaltene are classified as the polar compound. Resin and asphaltene are considered as surface active components which can act as a natural surfactants present in the crude oil/aqueous system, thus affecting the IFT.^31^ The acid number correlates the concentration of surface active components and is considered as an acidic crude oil if it is higher than 0.5 mg KOH per g.^32^

Crude oil used in this study was dead crude oil and was procured from the Cambay basin of ONGC. Characterization of crude oil was done with the help of SARA and Fourier Transform Infrared spectroscopy (FTIR) analysis. The physical properties of crude oil are shown in Table S1 (ESI†). The acid number of crude oil was measured as per ASTM D664 and SARA analysis was done according to the procedure detailed in ASTM D2007. It can be said from experimental data that the crude oil is acidic in nature. The other polar components with phenolic, carboxyl, amine and amide functionalities are also present in crude oil as evidenced from FTIR analysis. A detailed description of functional groups present in crude oil is shown in Fig. S1 (ESI†). A closer examination shows that crude oil mainly consists of phenolic, carboxyl, amine and amide functionalities along with some other groups.

### 2.2 Characterization of core sample

Mineralogical composition of rocks plays a significant role in controlling the mechanisms of LSWF, hence its characterization is of utmost importance while planning for LSWF for any oil reservoirs.^6,17^ The reservoir cores were characterized with the help of X-ray diffraction (XRD) and is shown in Fig. S2 (ESI†). It can be seen that the rock sample is mainly composed of calcite. The rock sample was powdered and XRD analysis of the powdered sample was done by X-ray diffractometer (D8 Advance Bruker). The mineralogical composition of the carbonate rock was analyzed with the help of standard library data (ICDD).

Semi-quantitative analysis was used to identify the mineralogy of carbonate rock by the Rietveld refinement method. It may be found that the rock is mainly composed of calcite mineral-79% and other minerals that are present in trace amount are quartz-4%, aragonite-12%, montmorillonite-3%, pyrite-2%. Carbonate reservoirs are generally found to be oil wet state. The initial oil-wet nature of the rock can be explained due to polar interactions, surface precipitation, acid/base interactions, and ion binding.^33^ Wettability alteration behavior of rocks is a complex phenomenon, which is dependent on interactions among oil/brine as well as rock/brine interfaces.^27^ Whilst changing the salinity of reservoir fluids, the extent and influence of the above-mentioned interactions weaken in order to cause gradual detachment of the crude oil and allow the injected brine phase to form a wetting film onto the rock surface.

### 2.3 Methodology and brine composition

All the brines were prepared with the help of deionized water by mixing the reagent grade salts (NaCl, MgCl_2_·6H_2_O, CaCl_2_·2H_2_O, NaHCO_3_, MgSO_4_, KCl and Na_2_SO_4_). Sea water was prepared by a standard procedure as mentioned in IS: 8770-1978 and further dilution of sea water were done with the help of deionized water. The formulation of sea water and its further dilution with other physical parameters are shown in Table S2 (ESI†). Similarly, to see the effect of ionic composition, ion tuned brine was also made and the formulation of ion tuned brine with different parameters are shown in Table S3 (ESI†). The brine solutions were filtered with a 0.45 μm filter paper to avoid any plugging.

**Fig. 1:**
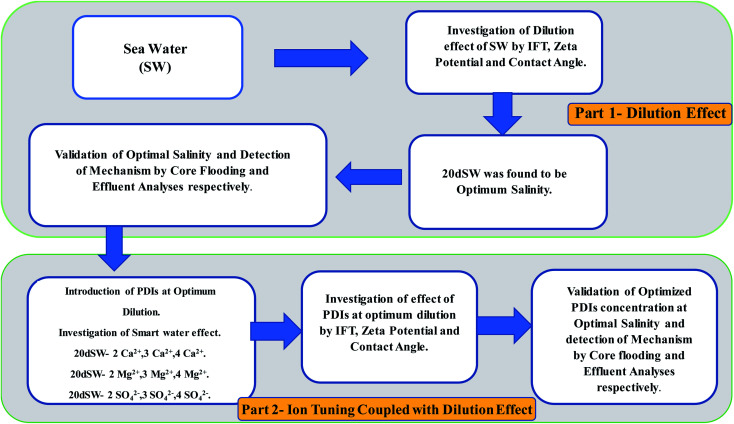
Proposed methodology of experiment.

The stepwise dilution of sea water and the alteration of ionic composition along with the methodology for the present study is shown in Fig. 1. A prior compatibility study of various brines with FW was also conducted. The injection of incompatible brine may lead to scaling/precipitation which will in turn decrease the permeability of reservoirs leading to formation damage. To check for compatibility, FW was mixed with various concerned brines in equal ratio and was kept in a dark container for one week under the reservoir temperature of 90 °C. Mixed brines were visually analyzed and no precipitation was observed.

### 2.4 IFT

The measurement of interfacial tension between the aqueous solution (different salinity and ionic composition) and crude oil was done by the pendant drop method with the help of drop shape analyzer. A video was recorded until the oil drop detaches from the J needle tip and the last image before detachment was used for IFT determination. IFT apparatus was calibrated with the standard procedure at water/air interface and a standard solvent heptane and water interface. Only after calibration, the measurements were taken. Experiments were repeated thrice. All the IFT measurements were done at 90 °C and atmospheric pressure.

### 2.5 Zeta potential measurement

Zeta potential was measured using Horiba scientific nano particle analyzer SZ-100. It measures the electrophoretic mobility of charged colloidal suspension. Samples of brine/rock were made according to the standard procedure. The solutions of brine/rock were prepared by adding 0.5 g of powdered rock into 50 ml of brine sample. Each solution was further sonicated for 3 min and left overnight to achieve equilibrium.^34^ After that, rock/brine solution was decanted and taken for measurement. For oil/brine zeta potential measurement, 0.5 ml of oil was mixed with respective 100 ml brine and sonicated for 3 minutes, left for some time and then taken for measurement.^35^ All the measurements were repeated 3 times for each sample. Also, the pH of each solution was measured as zeta potential is pH dependent. All the measurements of zeta potential and pH were made at 25 °C.

### 2.6 Contact angle measurement

For contact angle measurement, small slices of core were cut from the carbonate cores. Slices were first polished with sand paper to make the surface smooth to minimize the hysteresis effect due to surface roughness on the contact angle. Then the slices of core were placed under vacuum for 24 h and then it was aged with formation brine for another 24 h, then the slices were put into the crude oil and aging was done for 4 weeks at reservoir temperature of 90 °C to restore the plate wettability.

After the aging process has been completed, the slices were placed into the respective brine and where preheated. After the preheating, a crude oil drop was injected onto the rock slice. To see the behavior of oil drop with respect to time, the sealed vessel was put into oven which was maintained at 90 °C. Contact angle measurement has been carried out at reservoir temperature (90 °C) and at atmospheric pressure. The impact of pressure on wettability is weak to negligible whereas impact of temperature is predominant.^36,37^ This contact angle measurement procedure has also been used by various other researchers.^29^ Digital photographs were taken at a specific time interval and the contact angle was analyzed with the help of drop shape analyzer 25 (KRUSS) with in-built software DSA 4 in manual mode method. The first measurement was taken after 2 h so as to have a stable system and it was reported as 0 h reading. The contact angles were measured from the denser phase *i.e.* from brine phase which showed that a decrease in contact angle means that rock slice is going towards less oil wet condition. The rock is considered to be oil wet if the contact angle is in between 110° to 180°, neutral wet if the angle is in between 70° to 110° and water wet if contact angle is in between 0° to 70°.^38^ Contact angle measurements were repeated twice for each type of brine to eliminate the effect of local mineralogy and for its reproducibility concern.

### 2.7 Core flooding

The core flooding instrument consists of a Hassler type core holder, displacement pump (Teledyne Isco), calibrated collector, differential pressure gauge, slug container and an oven, schematic of which is shown in Fig. S3 (ESI†). The core holder was placed into the oven to stimulate the reservoir temperature and the displacement pump was used to inject the fluid with constant rate stored in a stainless-steel container. Injection fluid was heated with the help of a heating jacket before entering into the core holder. Overburden pressure of 2000 psi was applied with the help of a hydraulic pump by the injection of hydraulic oil between the rubber sleeve encasing the core and core holder's internal surface. A sample collector was used to collect the effluent samples. Carbonate cores were dried at 120 °C until the constant weight was achieved. Then the cores were saturated by formation water and left for 1 week so that ionic equilibrium could be achieved. Water permeability of cores were determined by injecting formation water at 90 °C with various injection rates of 0.5, 1.0, 1.5 and 2 ml min^−1^. The pressure drop across each core were monitored by digital pressure gauge until a steady state condition was reached and the permeabilities were calculated using Darcy's law. To establish the connate water saturation, dead crude oil was injected at a rate of 0.2 ml min^−1^ until only oil is produced at the outlet and no more water was produced. Then cores were kept in a sealed vessel filled with the crude oil in an oven for 4 weeks at a temperature of 100 °C. Table S4 (ESI†) shows the petrophysical properties of the core plugs used in this study. Core was remounted in the core holder for water flooding test and the fluid was injected in secondary mode at a flow rate of 0.2 ml min^−1^. All the experiments were conducted at a temperature of 90 °C and an overburden pressure of 2000 psi. Effluents were collected throughout the waterflooding experiment using the collector and the pressure drop was monitored with the help of differential pressure gauge installed across the core holder. Effluent collected was used to determine the oil recovery and ionic composition of produced brines.

### 2.8 Effluent analysis by ion-tracking

The produced effluent by core flooding was collected to conduct effluent's ionic analysis of active ions (SO_4_^2−^, Ca^2+^, Mg^2+^) with Metrohm ion chromatograph to gain a better insight into the mechanisms involved. The samples were first diluted with Millipore water and then filtered with a 0.02 μm syringe filter. Graphs were plotted as the ratio (*C*/*C*_0_) of produced concentration (*C*) of effluent PDIs and injected concentration (*C*_0_) of PDIs. Effluent analysis was carried out after some time the breakthrough has taken place.

## 3. Results and discussions

This section is discussed in two parts as also shown in Fig. 1. In the first part, the dilution effect of sea water has been discussed with the help of measurement of IFT, contact angle and zeta potential. An optimum salinity was obtained by analysis of rock/fluid and fluid/fluid interactive properties. In the second part, the effect of PDIs on optimum dilution was evaluated by IFT, contact angle and zeta potential measurements. Finally, the core flooding experiments were performed to check the efficacy of the designed ion tuned water for enhanced oil recovery. The analysis of the ionic composition of PDIs of the effluent was done to unlock the mechanisms underneath the increased oil recovery.

### 3.1 Effect of dilution of sea water (SW)

#### 3.1.1 Effect of dilution of sea water on interfacial tension (IFT)

**Fig. 2:**
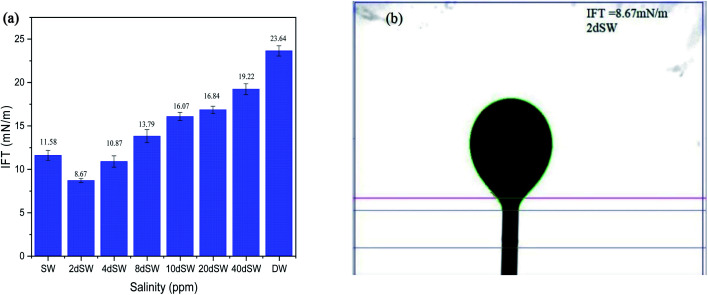
(a) IFT effect on dilution of SW (b) pendant drop image of lowest IFT at 90 °C.

The interfacial tension between crude oil and brine depends on various factors such as temperature, pressure, salinity, acid number and asphaltene content of crude oil.^39^ Interfacial properties between the brine and crude oil phase affects the oil recovery in LSWF. Hence, it is important to understand the chemical reactions at brine/oil interface due to the presence of divalent and monovalent ions in seawater and diluted sea water. The effect of dilution of the SW on IFT is shown in Fig. 2a. It can be seen from the figure that for the given crude oil and sea water with its subsequent dilution, the value of IFT first decreases with dilution and then starts to increase, indicating a salinity at which the value of IFT is minimum. Minimum value of IFT is obtained at 2dSW. A similar trend was also observed by Vijapurapu and Rao (2003).^40^

#### 3.1.2 Effect of dilution of SW on zeta potential

Zeta potential is mainly used to quantify the surface and electro kinetic phenomena at rock/brine and oil/brine interfaces. Charges on rock/brine and oil/brine are the basic entity that governs the stability of aqueous water film on the rock surfaces and hence the rock wettability. The stability of the water film is dependent on the strength of electrical interactions existing in-between oil, brine and rock.^41^ This charge on the interfaces can be discerned to predict the impact of rock/brine and oil/brine interactions on disjoining pressure. Disjoining pressure is a combination of three forces namely structural, electrostatic and van der Waals forces. If the net resulting balance of force is repulsive, then disjoining pressure is higher and then two interfaces (rock/brine and oil/brine interface) will be pushed apart thus creating thick water film. Thickness of water films decides oil wet and water wet nature of rocks.^42,43^

**Fig. 3:**
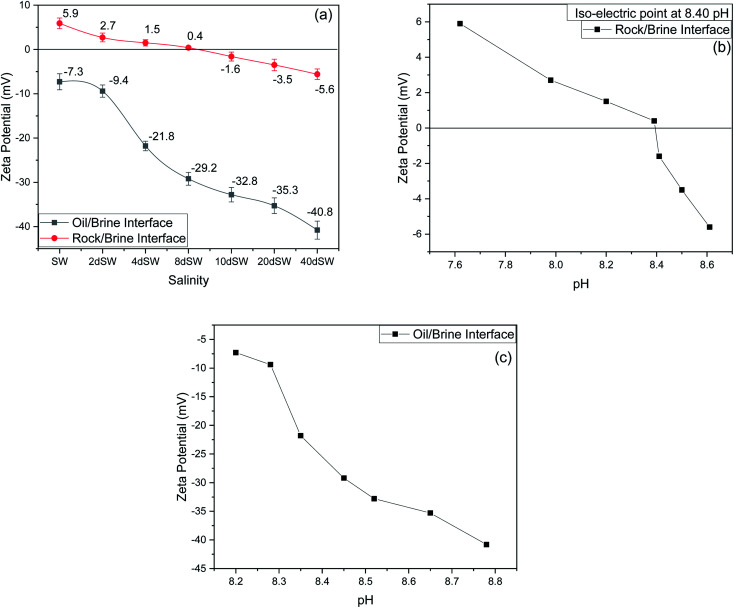
(a) Effect of salinity on zeta potential. (b) Effect of pH on zeta potential at rock/brine interface with salinity. (c) Effect of pH on zeta potential at oil/brine interface with salinity at 25 °C.

It can be seen from the results of zeta potential, as shown in Fig. 3a, that initially, the rock/brine interface was positively charged in sea water but as we dilute the sea water, the positive charge on the rock surface starts to shift to more negative, causing the double layer to expand. The value of zeta potential of carbonate rock dispersed in SW is positive,^29^ which is because of the abundant availability of calcite in carbonate rock and the value found is very close to the results reported by Alotaibi *et al.*^44^ Carbonate rock mainly constitutes of calcite, which is positively charged at neutral pH conditions.^45–47^ From literature, it is observed that the isoelectric point (IEP) of calcite surface is in between the pH value of 8.0 to 8.5 ^46,47^ and from our experimental result as shown in Fig. 3b, the isoelectric point is observed at pH of 8.40 which is in good agreement with literature. Charges present on calcite surfaces are found to be dependent on the isoelectric point. In this study, the charge at rock/brine interface are found to be positive at high salinity and pH lower than isoelectric point. Upon reducing the salinity and above the isoelectric point the zeta potential became negative. Hydrolysis reaction of carbonate in water generates the electric charge (mainly H^+^ and OH^−^ ions). Therefore, surface charge is pH dependent and can be positive and negative. Following are the general reaction (eqn (1)–(5)) that takes place when calcium carbonate is dissolved in water

1 CaCO_3(s)_ = CaCO_3(aq.)_

2 CaCO_3(aq.)_ = Ca^2+^ + CO_3_^2−^

3 CO_3_^2−^ + H_2_O = HCO_3_^−^ + OH^−^

4 Ca^2+^ + HCO_3_^−^ = CaHCO_3_^+^

5 Ca^2+^ + OH^−^ = CaOH^+^

At high pH concentration of negative species (HCO_3_^−^ and CO_3_^2−^) are high while at low pH positive species (Ca^2+^, CaHCO_3_^+^ and CaOH^+^) are higher leading to negative and positive charge respectively. At higher salinity, concentration of divalent ions is high due to which double layer is in compressed state. Upon dilution, less availability of divalent ions and high pH of calcite/water facilitates the negative species to realign themselves into the double layer and changes the surface charge. From the above discussion, we can say that below IEP, *i.e.*, at lower pH value the surface charge will be positive and above IEP at higher pH values, surface charge will be negative. It is observed from the literature that change in polarity of zeta potential affects the wettability and subsequent oil recovery.^48^ It can be also be seen from Fig. 3a that the zeta potentials of oil/brine interface are negative in charge regardless of dilution. As dilution takes place, the negative charge at the oil/brine interface is found to be increasing in magnitude. The pH of low salinity brine/oil interface as shown in Fig. 3c ranges from 8.2–8.8, which is also in agreement with literature that brine/oil zeta potential is negative at pH greater than 3.^49^ It is also worth noting that as dilution takes place, the charges on oil/brine interface leads to higher stability. This can be an indicative measure for the emulsification at oil/brine interface and hence transportation of oil in the form of stable emulsion, leading to enhanced oil recovery. Negative charges on both the surfaces are very much desired as it also ensures the positive effect of LSWF. It is also worth noting that the water film will be more stable if there is a repulsion between the two interfaces. Here, from Fig. 3a, we can see that up to 8dSW the charge on the rock/brine interface is positive which will lead to attraction between the rock/brine and oil/brine interfaces leading to unstable water film, thus rendering the rock more oil wet. After the 8dSW, charges on the rock/brine interface became negative with dilution, which will lead to repulsion and a more stable water film for wettability alteration. From the results, it can be concluded that the dilution of sea water after a certain level has positive effect of LSWF, leading to a more stable water film. From the above experimental results, it is clear that potential candidates for wettability alteration are 10dSW, 20dSW and 40dSW.

#### 3.1.3 Effect of dilution of sea water on wettability alteration

**Fig. 4:**
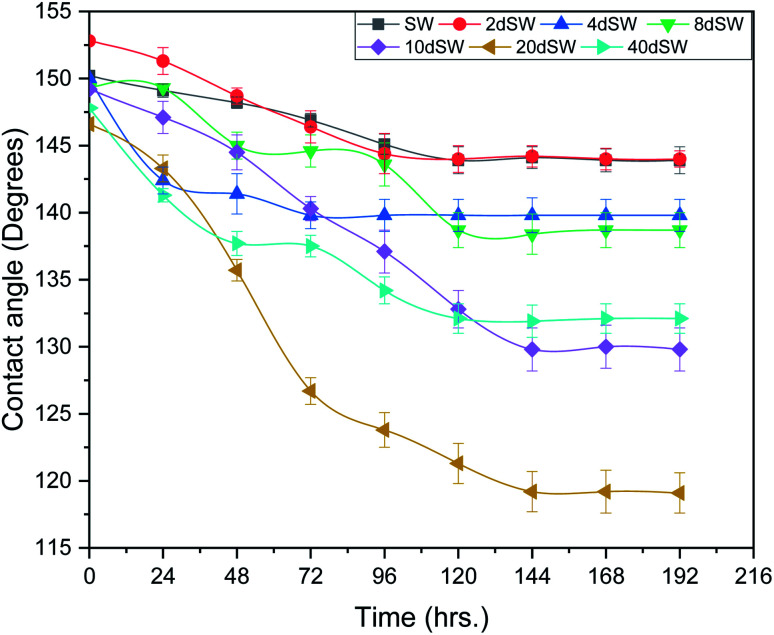
Effect of salinity on contact angle at 90 °C.

Wettability alteration is so far the most widely accepted mechanism for enhanced oil recovery by LSWF especially in carbonate reservoirs. Wettability alteration is mainly caused by the complex interaction that takes place between rock/brine/oil surfaces and their interactions are mainly governed by the mineralogy of rock, properties of brines and oil composition.^33,50^ The change of contact angle with respect to time and dilution of sea water was measured to ascertain the ability of dilution effect on the wettability alteration of oil-wet carbonate rock. It can be seen from Fig. 4, that the change in contact angle increases *i.e.*, contact angle decreases significantly with the degree of dilution of sea water. It can be seen that 10dSW, 20dSW and 40dSW showed a higher change in contact angle with respect to others which is well in agreement with the results found in zeta potential values as described above. Among the 10dSW, 20dSW and 40dSW, the higher change in contact angle was found to be with 20dSW which is considered as optimum salinity for further experiments. 40dSW showed less wettability alteration than 20dSW this may be due to lesser PDIs available in the solution. A detail discussion has been done on subsequent section. Images of time variant contact angle of optimum salinity are shown in Fig. S4 (ESI†). The determination of optimal salinity in LSWF is very challenging and it depends on rock mineralogy and composition of reservoir fluids and injection water.^16,17^ It was also argued that the maximum recovery of oil was found to be at optimal salinity.

#### 3.1.4 Core flooding and effluent ionic analysis of dilution effect

Core flooding experiments were performed at 90 °C with sea water (SW) and diluted seawater (20dSW and 40dSW) which show better properties as evidenced from the zeta potential and wettability alteration studies with respect to the reference SW. Although 20dSW is chosen to be the optimum salinity, core flooding was also done on 40dSW to test and verify our optimum salinity level. Effluents collected from core flooding were analyzed to get the insight of mechanisms that takes place in increased oil recovery through the dilution effect.

**Fig. 5:**
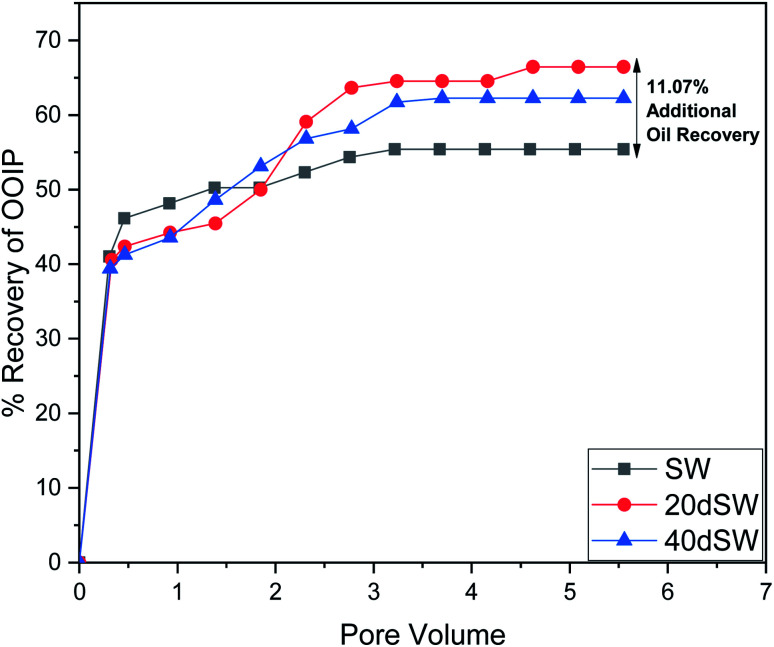
Oil recovery diagram for injection of SW, 20dSW and 40dSW in secondary mode at 90 °C.

**Fig. 6:**
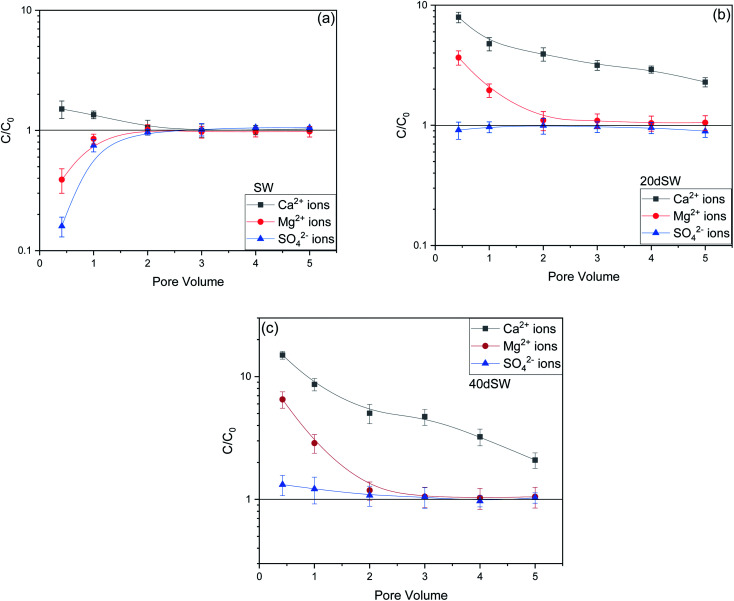
Effluent analysis of (a) SW (b) 20dSW (c) 40dSW.

**Fig. 7:**
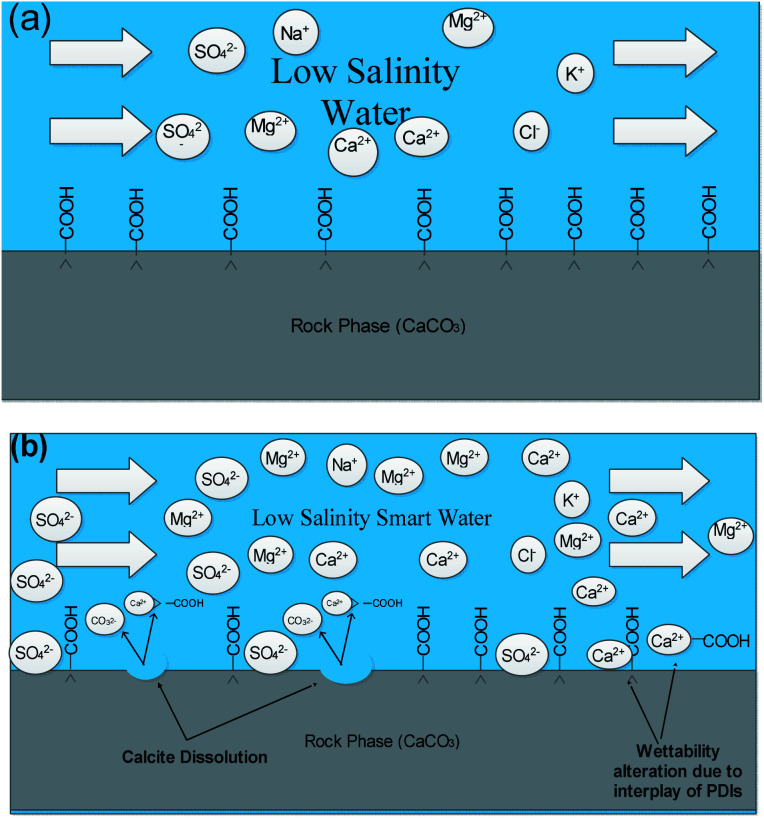
Schematic mechanisms prevailing at 20dSW oil recovery (a) injection of low salinity water (b) calcite dissolution and interplay of PDIs taking place.

From the core flooding experiment, it can be clearly seen that the dilution of sea water has a positive impact on improved oil recovery up to a certain threshold salinity level. It can be seen from Fig. 5 that injection of SW in carbonate reservoirs in secondary mode provides a recovery of 55.38% of OOIP whereas the injection of 20dSW results a recovery of almost 66.45% of OOIP and 40dSW produces a recovery of 62.20% OOIP which was less than the recovery obtained at optimum salinity level, thus confirming our optimum salinity hypothesis in dilution of SW. Injection of optimum salinity SW produced 11.07% additional oil recovery compared to the flooding of SW alone. It is very interesting to note that 40dSW showed an increased oil recovery than the SW flooding which was due to low salinity effect. But the recovery is less than 20dSW, which may be due to the lesser concentration of potential determining ions. It is also worth noting that the LSWF effect started to act after 1.5–2.0 PV. This delay in the LSWF effect suggests that LSWF is not instantaneous in nature rather it is very much time dependent phenomenon. The effluent analysis as shown in Fig. 6, provides some important light on the dominant mechanisms for increased oil recovery due to the dilution effect. From Fig. 6a, it can be seen there is not much appreciable change in the ionic concentration of SW effluent which suggests that the marginal oil recovery by SW is because of applied viscous force. In the case of 20dSW and 40dSW, there is an appreciable change in the concentration of PDIs, which suggests that increased oil recovery is assisted by some other mechanism leading to wettability alteration. From Fig. 6b, it can be seen that the concentration of Ca^2+^ ions is higher than that of the injection brine. It shows that Ca^2+^ ions are being supplied to the brine, due to the calcite dissolution. As dilution takes place there is a reduction in the ions present in the brine. When the injection of diluted brine takes place, it disturbs the pre-existing equilibrium prevailing with the high salinity brine. Thus, ions from rock dissolve into the injected brine. During the process, the dissolution of rock is accompanied by the release of adsorbed polar components of crude oil, thus increasing the water wetness of rock and hence thereby increased oil recovery. A schematic explaining the above mechanism is shown in Fig. 7. Calcite dissolution takes place according to following eqn (6) and (7) and is also supported by many authors.^4,8,51–53^

6 CaCO_3_ → Ca^2+^ + CO_3_^2−^

7 CO_3_^2−^ + H_2_O → HCO_3_^−^ + OH^−^

From Fig. 6b, it can also be seen that SO_4_^2−^ ions are getting adsorbed onto the rock surfaces which indicates that another mechanism that could be taking place simultaneously is wettability alteration due to interplay of PDIs. Due to this, SO_4_^2−^ ions gets adsorb onto the carbonate surfaces thus decreasing the positive surface charge density which in turn minimizes the electrostatic repulsive force between the positively charged carbonate surface and cations present in the brine. Due to this, co-adsorption of Ca^2+^ and other cations takes place to the surface. The Ca^2+^ ions thus react with the carboxylic acid groups that are bonded with the rock surface and thereby breaking the interactive interaction between oil and rock surface. This interaction of Ca^2+^ ions with carboxylic acid components lead to the formation of Ca^2+^–carboxylate complexes and their subsequent release from the rock surface alters the rock surface to a more water wet condition. For 40dSW, the same trend of effluent analysis has been observed indicating the calcite dissolution taking place except for the SO_4_^2−^ ions, which shows no variation indicating no interplay of PDIs. This could be due to the less availability of PDIs at 40dSW level and could be a reason for less recovery than 20dSW in which both the mechanism *i.e.*, calcite dissolution and interplay of PDIs leading to wettability alteration is taking place. From the Fig. 6b and c, it has been observed that the initial concentration of Mg^2+^ ion at breakthrough is higher compared to the injection water (20dSW and 40dSW) which is because of production of FW with high Mg^2+^ ions at the initial stage of flooding. Increased recovery due to LSWF is also because of the fact that crude oil is bonded by the organometallic complex between the polar component of crude oil and ions present in the formation water. The low salinity brine breaks these complexes and thus releases the crude oil and ions into the low salinity water being injected. The breakdown of these complexes by low salinity waterflooding is also believed to be big reasons for improved oil recovery. The results obtained were found to be in line with the conducted zeta potential and contact angle experiments. Although zeta potential gave three candidates for optimum recovery, it was the contact angle study that clearly indicates an optimum salinity for improved oil recovery. The mechanism that clearly dominates the oil recovery due to dilution of SW is calcite dissolution along with interplay of PDIs which led to wettability alteration of rock surface. Here, zeta potential acted as a screening criterion for increased oil recovery by dilution of SW in carbonate reservoirs.

### 3.2 Effect of potential determining ions (PDIs)

In this section, an effort has been made to see the effect of tuning the ionic composition in symbiotic association with the dilution effect of sea water. Basically, in this section we have tried to see the effect of PDIs (Ca^2+^, Mg^2+^, SO_4_^2−^) by varying their concentration at the optimum value of dilution *i.e.*, 20dSW that has been obtained from dilution approach earlier in this section. The effect was evaluated with the help of basic techniques such as IFT, contact angle and zeta potential measurements.

#### 3.2.1 Effect of optimum diluted sea water with varying PDIs on interfacial tension (IFT)

**Fig. 8:**
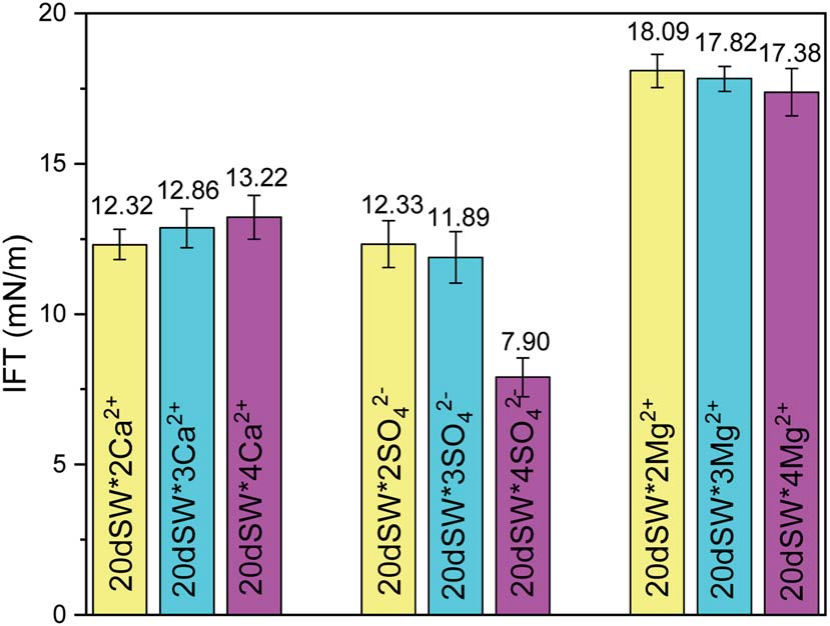
Ion tuning effect of PDIs on IFT at optimum dilution.

It has been observed in our study that the IFT didn't play an important role in deciding the optimum salinity obtained upon salinity variation. Further, the IFT of ion tuned water obtained through the variation of PDIs at optimum dilution were also checked for any significant IFT reduction. As seen in Fig. 8, all the brines lead to an increment in IFT values as compared to the IFT value of SW which is 11.58 mN m^−1^ as shown in Fig. 2a, except 20dSW × 2SO_4_^2−^ brine which lead to a reduction in IFT value of 3.68 mN m^−1^ only as compared to SW which can be considered as insignificant for EOR purposes. Thus, it may be concluded that IFT is not the leading mechanism in present study.

#### 3.2.2 Effect of optimum diluted sea water with varying PDIs on zeta potential

Proper tuning in the concentration of potential determining ions in injection brine could lead to a higher recovery by engendering the desired charges at rock/brine and oil/brine interfaces at a constant ionic strength of the solution. In this section, we have used 20dSW as base brine at constant ionic strength, to see the effect of potential determining ions on zeta potential and hence wettability alteration of rock samples. Concentrations of individual PDI were varied to 2 times, 3 times and 4 times the concentration that is present in 20dSW keeping the concentration of other PDIs constant. These types of brine are termed as ion tuned water as shown in Table S3 (ESI†).

**Fig. 9:**
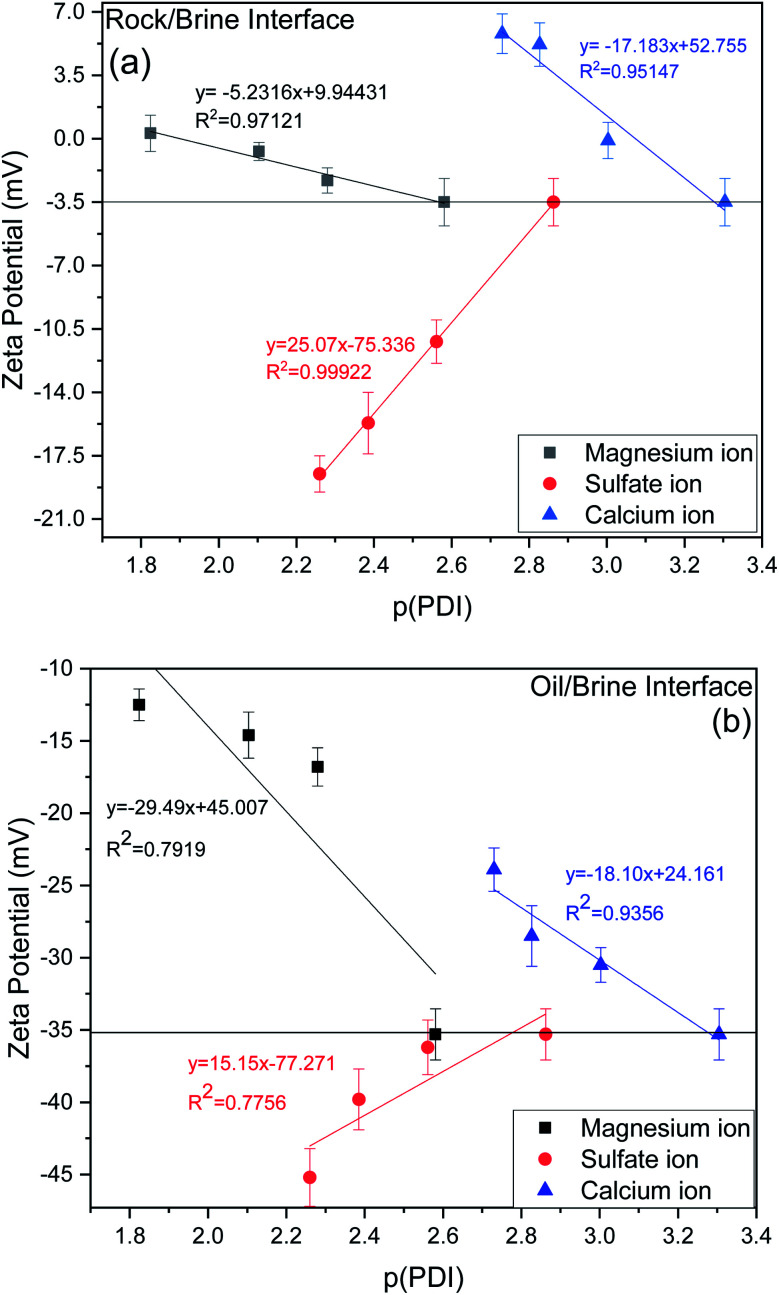
Variation of zeta potential (a) rock/brine interface (b) oil/brine interface of ion tuned brine at optimum dilution of 20dSW at 25 °C.

From experimental data as shown in Fig. 9a for the rock/brine interface, it can be observed that increasing concentration of calcium and magnesium in the 20dSW, increase the magnitude of zeta potential at the rock/brine interface towards the more positive side which is due to the increased affinity of these ions toward the carbonate rock surface. Thereby making the surrounding surface more positively charged and hence shifting the charges present at rock/brine surface towards more positive side. Here *p*(PDI) represents the negative logarithm of PDI concentration in mol L^−1^ and decreasing *p*(PDI) values means there is an increase in the concentration of respective PDI. It can be seen that as *p*Ca value decreases, the zeta potential becomes more positive which is consistent with increased adsorption of Ca^2+^ onto the calcite surfaces.^54^ A close to linear relationship is found between zeta potential and *p*Ca, which is consistent with Nernstian behavior of calcite surface and suggests that the electrical double layer can be reasonably described by the Gouy–Chapman–Grahame model close to the isoelectric point. Similar results are also observed in the case of Mg^2+^ which also shows a close to linear relationship with respect to decreasing *p*Mg concentration.^12,55,56^ It can be seen that both Ca^2+^ and Mg^2+^ ions behaves similarly with given carbonate rock surface.^55^ Apart from this, an opposite trend has been observed in the case of SO_4_^2−^ ions. The charge on the rock surface was increasing towards the more negative side with the increasing concentration of SO_4_^2−^ ions, because of increased affinity of SO_4_^2−^ ions towards the rock surface due to which rock surface becomes more negatively charged.

For designing the optimum ionic concentration for the waterflooding process, it is very important to know the effect and charges of both rock/brine and oil/brine interfaces so that the positive effect of low salinity waterflooding can be harnessed.^27^ Although many studies have been conducted for quantifying the charges present at rock/brine interface with varying PDIs,^11,12,28^ the investigation on the variation of charges present at the oil/brine interface with varying PDIs needs to be explored in detail. For this purpose, the variation of zeta potential at oil/brine interface with varying PDIs are also studied and is shown in Fig. 9b. It can be seen that the addition of both the ions individually *i.e.* Ca^2+^ and Mg^2+^, decreases the negative charges at the oil/brine interface. This may be due to the interaction of carboxylic acid compounds present at the oil/brine interface with the gradually increasing concentration of Ca^2+^ and Mg^2+^ ions in the brine solution. The reduction in charge is found to be less upon the addition of Ca^2+^ ions than the addition of Mg^2+^ ions. This is due to the difference in charge density of the cations. Ca^2+^ ions are having less charge density and are weakly hydrated than the Mg^2+^ ions. Thus, it requires less entropy change resulting in favorable adsorption. This led to the formation of a stable Ca^2+^–carboxylate complexes at the interface leading to less reduction in charges and locking of the acidic component of oil at interface, leading to a stable emulsion system for increased oil recovery. Mg^2+^ ions possess high charge density and their strong hydration contributes to the repulsion from the oil/brine interface.^57,58^ Ca^2+^ and Mg^2+^ ions were found to follow the Hofmeister series for a stable emulsion formation where Ca^2+^ ions form a stable emulsion than Mg^2+^ ions also indicated by charges present at the interface as shown in Fig. 9b. An opposite trend is shown by the SO_4_^2−^ ions, it can be seen that upon increasing the SO_4_^2−^ ions concentration, zeta potential at oil/brine interface shifts to a more negative side. This is due to the adsorption of SO_4_^2−^ ions onto the oil/brine interface with the help of positively charged amide group present in the crude oil, as evident from FTIR of crude oil, shown in Fig. S1 (ESI†). Presence of carboxylic acid and SO_4_^2−^ ions lead to an increase in the magnitude of negative charge at the oil/brine interface upon the addition of SO_4_^2−^ ions. Zeta potential is dependent on pH, so it is imperative to discuss the effect of pH. Very few studies have shown the effect of pH due to variation of PDIs.^54^ The rock/brine solution pH of sulfate (8.44–8.50) and magnesium (8.66–8.70) did not vary much as the zeta potential of calcite is independent of pH if the concentration of calcium is kept constant.^59,60^ Also, the solution pH of Ca^2+^ ions varied insignificantly from 8.55 to 8.25. Since the pH was almost constant for each ion tuned water, so it can be inferred that the variation in zeta potential values are due to the ionic tuning of PDIs only. Thus, from the above results it can be said that, the tuning of PDIs in the solution has good potential to alter the charges of rock/brine and oil/brine interfaces leading to engineered interfaces for increased oil recovery.

#### 3.2.3 Effect of PDIs on wettability alteration of carbonate rocks

In this section, an effort has been made to see the effect of ion tuned water on rock/oil/brine three phase contact angle and its impact on ion tuned water flooding. Contact angle was measured for all the brine having an enhanced concentration of PDIs. Change in contact angle was evaluated to ascertain the enhanced modification in wettability alteration by ion tuned brine as compared to the dilution effect.

**Fig. 10:**
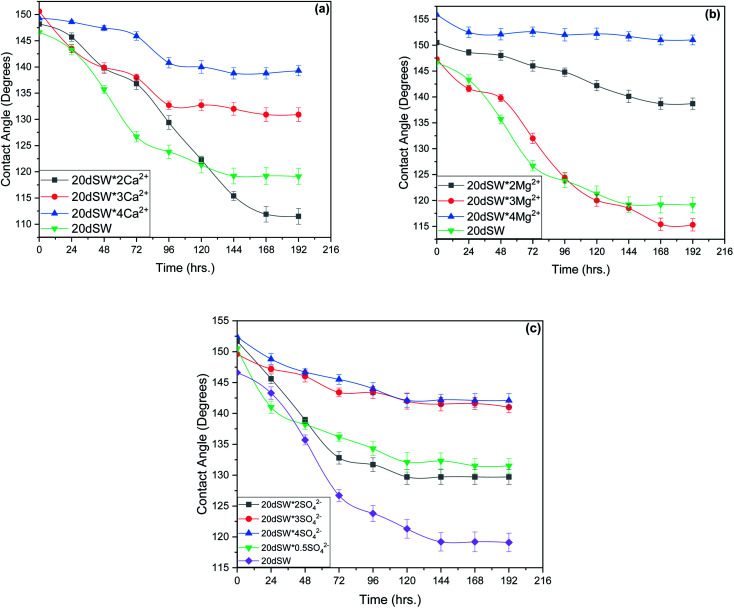
Time variant change in contact angle at 90 °C for (a) Ca^2+^ ion tuning (b) Mg^2+^ ion tuning and (c) SO_4_^2−^ ion tuning.

From the experimental study as shown in Fig. 10, it is clear that there exists an optimum value of PDIs concentration obtained upon the tuning of optimum diluted seawater (20dSW). In case of Ca^2+^ ion tuning, optimum value is obtained at 20dSW × 2Ca^2+^ and for Mg^2+^ ion tuning, the optimum value is obtained at 20dSW × 3Mg^2+^. The effect of SO_4_^2−^ ion tuning on wettability alteration is complex as shown in Fig. 10c. Actually, the role of SO_4_^2−^ ion is different from that of Ca^2+^ and Mg^2+^ in ion tuned water flooding. The SO_4_^2−^ ions are preferentially adsorbed on the positively charge carbonate rock, which in turn displaces the polar oil components and makes the surface more water-wet. As the SO_4_^2−^ ions get adsorbed on the carbonate rock, its positive surface charge is decreased and consequently more Ca^2+^ gets attracted to the surface due to less electrostatic repulsion. Then Ca^2+^ removes the adsorbed polar oil component from the surface according to the following reaction as proposed by Rezaeidoust *et al.*:^20^

8 RCOO^−^–Ca–CaCO_3_ (s) + Ca^2+^ + SO_4_^2−^ = RCOO–Ca^+^ + Ca–CaCO_3_ (s) + SO_4_^2−^

Similarly, the oil detachment mechanism of Mg^2+^ in presence of SO_4_^2−^ is shown by eqn (9). In both the cases SO_4_^2−^ catalyzes the reactions.

9 RCOO^−^–Ca–CaCO_3_ (s) + Mg^2+^ + SO_4_^2−^ = Mg–CaCO_3_ (s) + RCOO–Ca^+^ + SO_4_^2−^

An optimum concentration of SO_4_^2−^ ion is required to achieve the maximum benefits by ion tuned water flooding beyond this concentration, the effect of addition of SO_4_^2−^ ion is found to be detrimental.^29^ The present study indicates that the SO_4_^2−^ ion concentration corresponding to 20dSW is the optimum as it offers the maximum wettability alteration manifested through contact angle compared to other concentrations as shown in Fig. 10c.

#### 3.2.4 Core flooding and effluent ionic analysis of ion tuned water

**Fig. 11:**
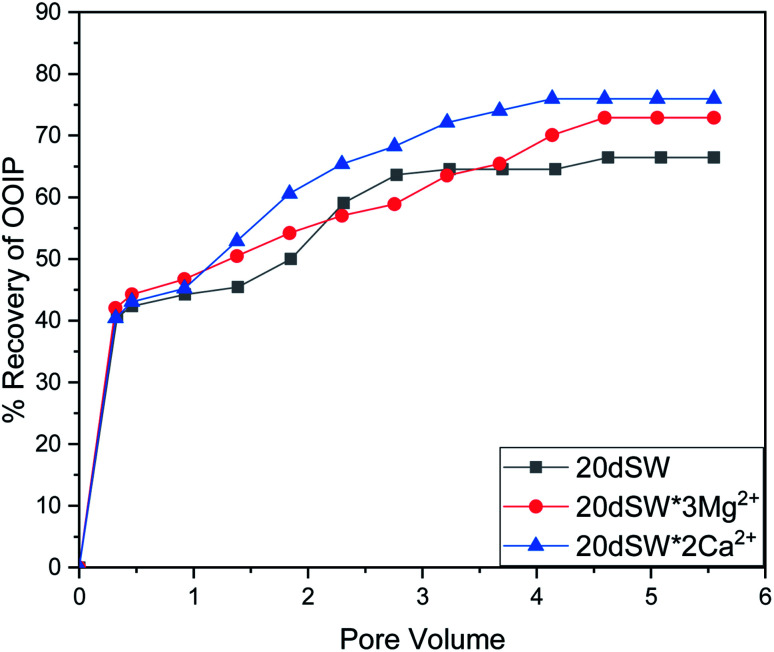
Oil recovery diagram for injection of 20dSW, 20dSW × 3Mg^2+^ ion and 20dSW × 2Ca^2+^ ion tuned brine in secondary mode at 90 °C (ion tuning effect coupled with dilution).

**Fig. 12:**
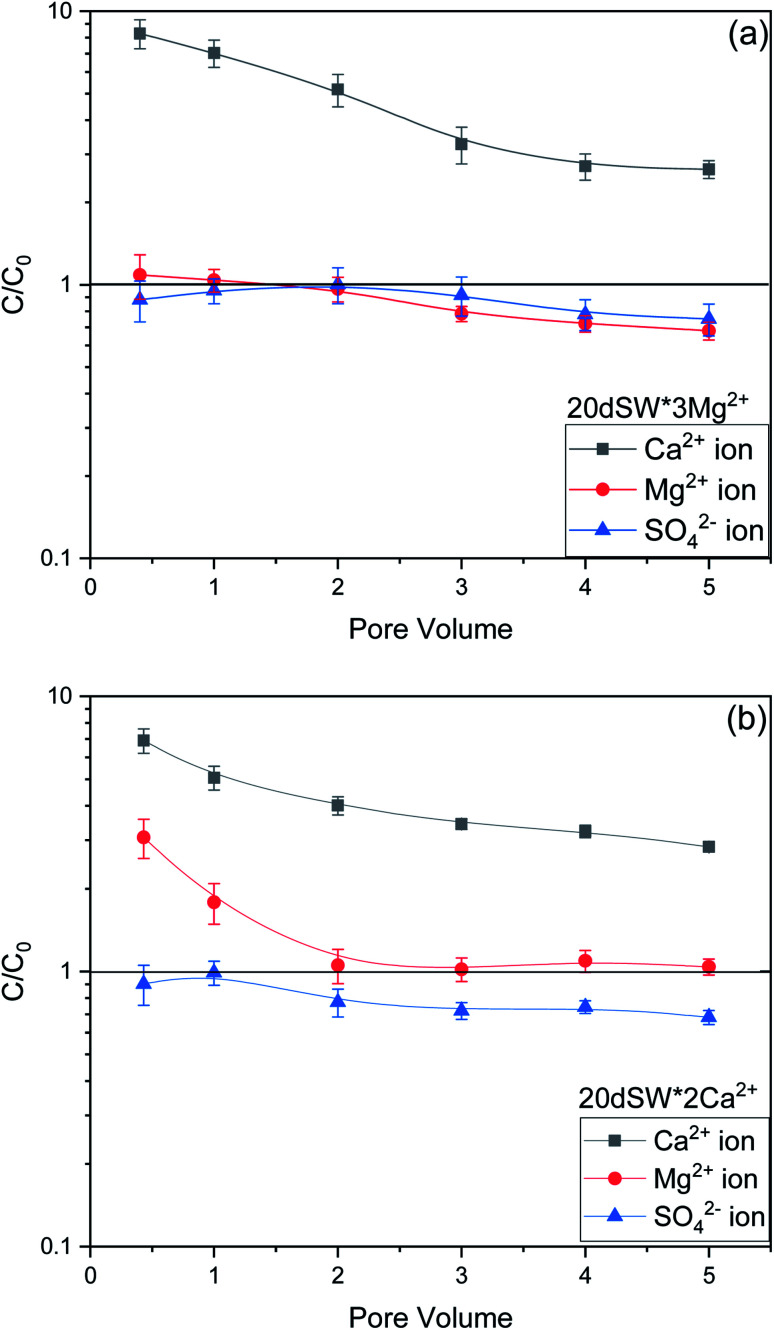
Effluent analysis of (a) 20dSW × 3Mg^2+^ (b) 20dSW × 2Ca^2+^.

To establish the mechanisms of enhanced oil recovery by ion tuned water flooding, the effluents collected after the flooding were analyzed to correlate with the recovery data. In the case of Mg^2+^ ions tuning, it can be seen that zeta potential results gave two potential candidates, namely 20dSW × 2Mg^2+^ and 20dSW × 3Mg^2+^ for enhanced wettability alteration. The charges of these ion tuned water on the rock/brine interface is on negative side as shown in Fig. 9a. Negative charges on rock/brine and oil/brine interface can lead to a stable water film and can have an enhanced effect on changing the wettability of rock. The contact angle measurements were considered to determine the optimum ionic concentration of Mg^2+^ ions. The brine with composition 20dSW × 3Mg^2+^ showed the enhanced wettability alteration compared to 20dSW as shown in Fig. 10b. Wettability alteration results show that tailoring the Mg^2+^ ions concentration at 20dSW can lead to a more recovery than SW and 20dSW. This was also confirmed with the core flooding results, as shown in Fig. 11 and mechanisms underlying for increased oil recovery were predicted based on effluent analysis as shown in Fig. 12a. Ca^2+^ concentration is significantly higher at breakthrough compared to its concentration in the injected water (20dSW × 3Mg^2+^). With the increase in pore volume of injected of water, the concentration of Ca^2+^ gradually decreases but it is still higher than the concentration in the injection water indicating calcite dissolution. Fig. 12a, also indicated the sulphate adsorption which can be accounted for the interplay of PDIs leading to wettability alteration as explained in Section 3.1.4. On the other hand, it has been observed that with an increase in PV of injected water, the concentration of Mg^2+^ falls below its initial concentration. The consistent increase in concentration of Ca^2+^ ions and decrease in the concentration of Mg^2+^ ion from the injected initial concentration indicates that dolomitization might be taking place at the rock surface during the low salinity water flooding. The dolomitization is mainly dependent on the concentration ratio of [Mg^2+^/Ca^2+^] ions, which increases in the case of Mg^2+^ rich brines. As the ratio of [Mg^2+^/Ca^2+^] ion increases, the intensity of dolomitization also increases. Also, the production of dolomite is accomplished more readily in diluted solution.^61^ But the production of dolomite is very slow phenomenon and requires large enough time to take place.^62,63^ So, the decrease in concentration on Mg^2+^ ion can be attributed to the adsorption of Mg^2+^ ions on the oil wet carbonate surfaces.

As discussed earlier, to have stable brine film between rock and oil, the charges both at rock/brine and oil/brine interfaces should be the same. From zeta potential result, it was found that as the concentration of Ca^2+^ was increased, zeta potential values shifted from negative to positive side as shown in Fig. 9a, whereas the zeta potential value on the oil/brine interface is negative as shown in Fig. 9b. To have positive effect of Ca^2+^ ion tuning, the zeta potential values should be on the negative side for the rock/brine interface. The negative charge on the rock/brine interface was found for 20dSW × 2Ca^2+^ brine only as shown in Fig. 9a. Contact angle experiments confirmed this result and thus offers an optimum salinity for Ca^2+^ ion tuning. The core flooding and effluent analysis of 20dSW × 2Ca^2+^ brine are shown in Fig. 11 and 12b respectively. The dominant mechanism proposed behind the increased oil recovery of 20dSW × 2Ca^2+^ is interplay of PDIs in combination with calcite dissolution, leading to wettability alteration of rock surfaces. The available SO_4_^2−^ ions get adsorbed onto the rock surfaces diminishing the positive charge present on carbonate rock to a larger extent, thus providing the chance for Ca^2+^ and Mg^2+^ ions to come and interact in the vicinity of attached crude oil components. Thus, the desorption of crude oil from the rock surface leads to increased oil recovery. A schematic of the above mechanism is also shown in Fig. 7. It has been observed that the initial concentration of Mg^2+^ ion at breakthrough is higher compared to the injection water (20dSW × 2Ca^2+^) which is because of production of FW with high Mg^2+^ ions at the initial stage of flooding and then slowly decreases due to subsequent injection of diluted ion tuned water and approaches to the original concentration of injection water. Among Ca^2+^, Mg^2+^ and SO_4_^2−^ ion tuning, maximum wettability alteration and subsequent oil recovery are found to be with 20dSW × 2Ca^2+^ brine which can be attributed to the higher activity of Ca^2+^ ions. At temperatures lower than 100 °C, Ca^2+^ ions have greater activity than the Mg^2+^ ions.^5,12,21,64^ Due to higher activity and increased concentration of Ca^2+^ ions, 20dSW × 2Ca^2+^ ion tuned water is able to detach more oil from the surface of carbonate reservoirs than Mg^2+^ ions brines. The Ca^2+^ and Mg^2+^ ions showed different optimum concentrations. The different response of Ca^2+^ and Mg^2+^ ions can be attributed to the activity of different ions which significantly varies with temperature. The addition of Ca^2+^ ions with increased activity, lead to a maximum recovery than the addition of Mg^2+^ ions. In case of Mg^2+^ ion addition, the Ca^2+^ ions were kept constant but at the same time the concentration of Na^+^ ions decreased significantly which ensured an unhindered activity of Ca^2+^ ions along with the Mg^2+^ ions. Since the activity of Mg^2+^ ions are less than Ca^2+^ ions at a temperature less than 100 °C this led to increased concentration of Mg^2+^ ions than the Ca^2+^ ions for an optimum value.

**Fig. 13:**
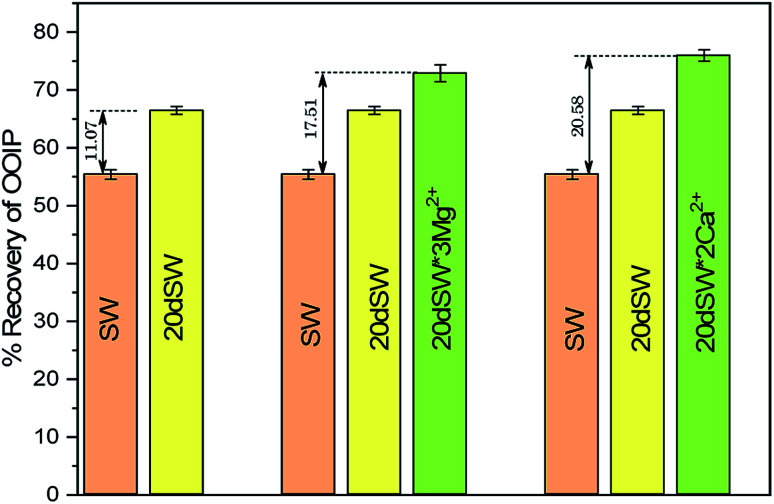
Comparison of oil recovery obtained both by dilution and ion tuned water flooding of SW, 20dSW, 20dSW × 3Mg^2+^ and 20dSW × 2Ca^2+^ brines.

The optimum salinity proposed for tailored ion tuned water was subjected to core flooding to validate the contact angle results for improved oil recovery. Core flooding with diluted SW and optimum diluted ion tuned water are shown in Fig. 5 and 11 respectively. SW and 20dSW showed a recovery factor of 55.38% and 66.45% OOIP, respectively. The brine 20dSW × 2Ca^2+^ showed the maximum recovery of 75.96% OOIP, 20dSW × 3Mg^2+^ showed maximum recovery of 72.89% OOIP. It can be seen that all the brines either obtained by dilution or dilution coupled with ion tuning showed improved oil recovery than the injection of SW. From the above discussions, it can be said that injection of different water recipe to the same reservoir can lead to different oil recovery. Thus, a comprehensive study is needed to optimize the oil recovery due to LSWF before injecting into the reservoir. A comparative graph of oil recovery by SW and all the optimized brines are shown in Fig. 13. From the figure, it is clear that both the approaches *i.e.*, dilution and ion tuning have its own benefit on increased oil recovery and can be combined to harness the enhanced potential for increased oil recovery. Overall in this study, calcite dissolution and interplay between PDIs leading to wettability alteration of rock to less oil wet condition, played the dominant role both in dilution and ion tuning which gave rise to higher improved oil recovery.

## 4. Conclusions

In this study, the potential of oil recovery by low salinity waterflooding was evaluated by combining the dilution and ion tuning approaches in carbonate reservoir. Both methods showed positive effect on oil recovery. Effect of sea water dilution was investigated and the properties obtained were improved by ion tuning of Mg^2+^ and Ca^2+^ ions. Core flooding was done on all the optimum brines obtained from both the dilution and ion tuning. Effluent analysis was done to gain insight into the underlying mechanisms. The following conclusions can be drawn from the performed study:

• Upon dilution of SW, charge present at carbonate surface at rock/brine interface shifted from positive to negative side.

• Contact angle studies upon dilution of SW showed that an optimum salinity at 20dSW exists.

• Core flooding conducted on 20dSW showed an increase of 11.07% of OOIP as compared to SW.

• Zeta potential studies conducted on ion tuned brines at optimum dilution showed that tuning of SO_4_^2−^, Mg^2+^ and Ca^2+^ ions almost linearly affected the surface charges at rock/brine interface and can lead to an engineered rock/brine interface.

• Core flooding conducted with 20dSW × 3Mg^2+^ and 20dSW × 2Ca^2+^ ion tuned water showed an increase in oil recovery of 17.51% and 20.58% of OOIP respectively over SW.

• Calcite dissolution and interplay of PDIs were found to be the dominating mechanisms and lead to wettability alteration of carbonate rock, which in turn improved the oil recovery.

## Conflicts of interest

The authors declare that they have no known competing financial interests or personal relationships that could have appeared to influence the work reported in this paper.

## Supplementary Material

RA-010-D0RA08301A-s001

## References

[cit1] Klemme H. D., Ulmishek G. F. (1991). Am. Assoc. Pet. Geol. Bull..

[cit2] Reeder R. J. , Carbonates: mineralogy and chemistry, Reviews in Mineralogy, Mineral. Soc. Amer., 1990, vol. 11

[cit3] Austad T. , Strand S., Madland M. V., Puntervold T. and Korsnes R. I., International Petroleum Technology Conference, IPTC-11370-MS, Society of Petroleum Engineers, 2007

[cit4] Chandrasekhar S. , Sharma H. and Mohanty K. K., SPE Annual Technical Conference and Exhibition, SPE-181700-MS, Society of Petroleum Engineers, 2016

[cit5] Al-Shalabi E. W., Sepehrnoori K. (2016). J. Pet. Sci. Eng..

[cit6] Mahani H., Keya A. L., Berg S., Bartels W. B., Nasralla R., Rossen W. R. (2015). Energy Fuels.

[cit7] Strand S., Henningsen S. C., Puntervold T., Austad T. (2017). Energy Fuels.

[cit8] Yousef A. A. , Al-Saleh S., Al-Kaabi A. U. and Al-Jawfi M. S., Can. Unconv. Resour. Int. Pet. Conf., SPE-137634-MS, 2010

[cit9] Zahid A. , Stenby E. H. and Shapiro A. A., SPE Europec/EAGE Annual Conference, SPE-154508-MS, 2012

[cit10] Strand S., Høgnesen E. J., Austad T. (2006). Colloids Surf., A.

[cit11] Zhang P., Austad T. (2006). Colloids Surf., A.

[cit12] Zhang P., Tweheyo M. T., Austad T. (2007). Colloids Surf., A.

[cit13] Karoussi O., Hamouda A. A. (2007). Energy Fuels.

[cit14] Purswani P., Karpyn Z. T. (2019). Fuel.

[cit15] Yousef A. A., Al-Saleh S. H., Al-Kaabi A., Al-Jawfi M. S. (2011). SPE Reservoir Eval. Eng..

[cit16] Zaeri M. R., Hashemi R., Shahverdi H., Sadeghi M. (2018). Pet. Sci..

[cit17] Nasralla R. A., Mahani H., van der Linde H. A., Marcelis F. H. M., Masalmeh S. K., Sergienko E., Brussee N. J., Pieterse S. G. J., Basu S. (2018). J. Pet. Sci. Eng..

[cit18] Austad T. , Strand S., Høgnesen E. J. and Zhang P., SPE Int. Symp. Oilf. Chem., SPE-93000-MS, 2005

[cit19] Fathi S. J., Austad T., Strand S. (2011). Energy Fuels.

[cit20] Rezaeidoust A., Puntervold T., Strand S., Austad T. (2009). Energy Fuels.

[cit21] Zhang P., Tweheyo M. T., Austad T. (2006). Energy Fuels.

[cit22] Zhang Y. and Sarma H., Abu Dhabi Int. Pet. Exhib. Conf., SPE-161631-MS, 2012

[cit23] Hiorth A., Evje S. (2010). Netw. Heterog. Media.

[cit24] Austad T., Shariatpanahi S. F., Strand S., Black C. J. J., Webb K. J. (2012). Energy Fuels.

[cit25] Austad T. , Enhanc. Oil Recover. F. Cases, Gulf Professional Publishing, Amsterdam, 2013, ch. 13, pp. 301–332

[cit26] Shehata A. M., Alotaibi M.
B., Nasr-El-Din H. A. (2014). SPE Reservoir Eval. Eng..

[cit27] Jackson M. D., Al-Mahrouqi D., Vinogradov J. (2016). Sci. Rep..

[cit28] Purswani P., Karpyn Z. T. (2019). Fuel.

[cit29] Awolayo A. , Sarma H. and AlSumaiti A. M., SPE EOR Conf. Oil Gas West Asia, SPE-169662-MS, 2014

[cit30] Aske N., Kallevik H., Sjo J. (2001). Energy Fuels.

[cit31] Havre T. E., Sjöblom J., Vindstad J. E. (2003). J. Dispersion Sci. Technol..

[cit32] Rana B. S., Cho D. W., Cho K., Kim J. N. (2018). Fuel.

[cit33] Buckley J. S., Liu Y., Monsterleet S. (1998). SPE J..

[cit34] Mahani H., Keya A. L., Berg S., Nasralla R. (2017). SPE J..

[cit35] Takeya M., Shimokawara M., Elakneswaran Y., Nawa T., Takahashi S. (2019). Fuel.

[cit36] Hjelmeland O. S., Larrondo L. E. (1986). SPE Reservoir Eng..

[cit37] Zhang Y., Zeng J., Qiao J., Feng X., Dong Y. (2018). Energy Fuels.

[cit38] Anderson W. G. (1986). J. Pet. Technol..

[cit39] Buckley J. S. , Fan T., Crude Oil/Brine Interfacial Tensions, Society of Petrophysicists and Well-Log Analysts, 2007, vol. 48, pp. 175–185

[cit40] Vijapurapu C. S. and Rao D. N., Int. Symp. Oilf. Chem., SPE-80273-MS, 2003

[cit41] Sari A., Xie Q., Chen Y., Saeedi A., Pooryousefy E. (2017). Energy Fuels.

[cit42] Myint P. C., Firoozabadi A. (2015). Curr. Opin. Colloid Interface Sci..

[cit43] Dubey S. T., Doe P. H. (1993). SPE Reservoir Eng..

[cit44] Alotaibi M. B., Nasr-El-Din H. A., Fletcher J. J. (2011). SPE Reservoir Eval. Eng..

[cit45] Somasundaran P., Agar G. (1967). J. Colloid Interface Sci..

[cit46] Anderson W. G. (1987). J. Pet. Technol..

[cit47] Pokrovsky O. S., Schott J., Thomas F. (1999). Geochim. Cosmochim. Acta.

[cit48] Al Mahrouqi D. A. , Vinogradov J. and Jackson M. D., SPE Latin American and Caribbean Petroleum Engineering Conference, SPE-177242-MS, 2015

[cit49] Takamura K., Chow R. S. (1985). Colloids Surf..

[cit50] Nasralla R. A. , Bataweel M. A., Nasr-el-din H. A. and Texas A., SPE Offshore Eur. Oil Gas Conf. Exhib., SPE-146322-MS, 2011

[cit51] Awolayo A. N., Sarma H., Nghiem L. X. (2018). Energy Fuels.

[cit52] Hamouda A. A., Maevskiy E. (2014). Energy Fuels.

[cit53] Hiorth A., Cathles L. M., Madland M. V. (2010). Transp. Porous Media.

[cit54] Al Mahrouqi D., Vinogradov J., Jackson M. D. (2017). Adv. Colloid Interface Sci..

[cit55] Alroudhan A., Vinogradov J., Jackson M. D. (2016). Colloids Surf., A.

[cit56] Chen L., Zhang G., Wang L., Wu W., Ge J. (2014). Colloids Surf., A.

[cit57] Dos Santos A. P., Levin Y. (2012). Langmuir.

[cit58] Taylor S., Chu H. (2018). Colloids Interfaces.

[cit59] Cicerone D. S., Regazzoni A. E., Blesa M. A. (1992). J. Colloid Interface Sci..

[cit60] Sondi I., Bišćan J., Vdović N., Škapin S. D. (2009). Colloids Surf., A.

[cit61] Folk R. L., Land L. S. (1975). Am. Assoc. Pet. Geol. Bull..

[cit62] Escorcia L.-C., Gomez-Rivas E., Daniele L., Corbella M. (2013). Geofluids.

[cit63] Hanshaw B. B., Back W., Deike R. G. (1971). Econ. Geol..

[cit64] Awolayo A., Sarma H., Nghiem L. (2018). Energies.

