# Supplementary material for: A mechanistic investigation of low salinity water flooding coupled with ion tuning for enhanced oil recovery
Source: RSC Adv. 2020 Nov 24;10(69):42570–83. doi: 10.1039/d0ra08301a (PMC9057959; doi:10.1039/d0ra08301a)
Supplement: RA-010-D0RA08301A-s001 [file RA-010-D0RA08301A-s001.pdf]

Electronic Supplementary Material (ESI) for RSC Advances.
This journal is © The Royal Society of Chemistry 2020

Electronic Supporting Information (ESI)

For the article:

A Mechanistic Investigation of Low Salinity Water Flooding Coupled with Ion Tuning for Enhanced Oil Recovery

Rohit Kumar Saw, Ajay Mandal *

Chemical Flooding Laboratory, Department of Petroleum Engineering

Indian Institute of Technology (Indian School of Mines), Dhanbad - 826004, India

*Corresponding Author, Email: ajay@iitism.ac.in

Table S1: Physical parameters of crude oil

API @15℃	23.40
Density (g/cm^3) @60℃	0.8849
Acid number (mg KOH/g)	0.6
Saturates (%)	52.9
Aromatics (%)	29.5
Asphaltenes (%)	7.8
Resins (%)	9.7
Viscosity (cp)	5.1

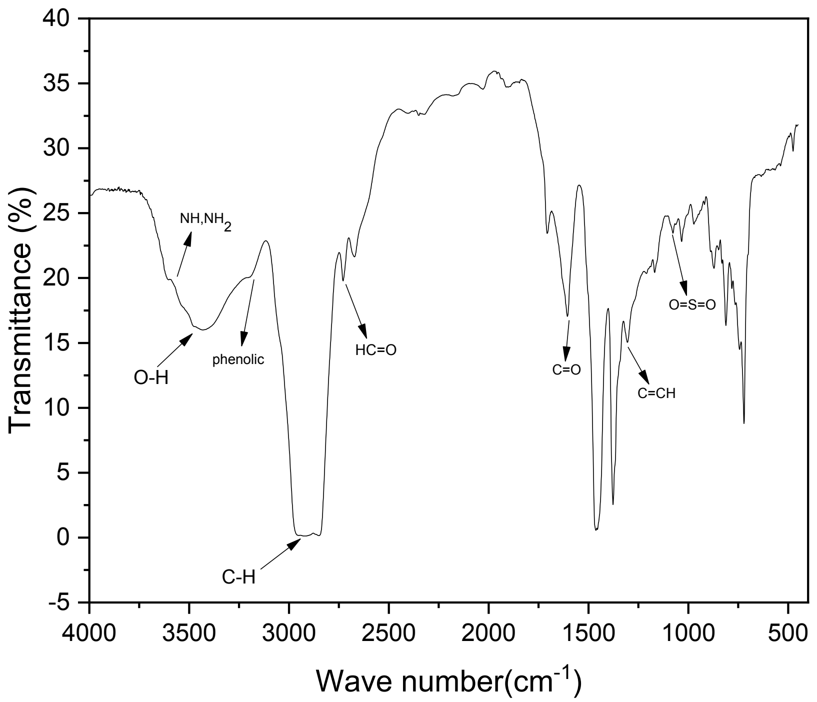

Fig. S1. FTIR Characteristics of Crude Oil used in this study

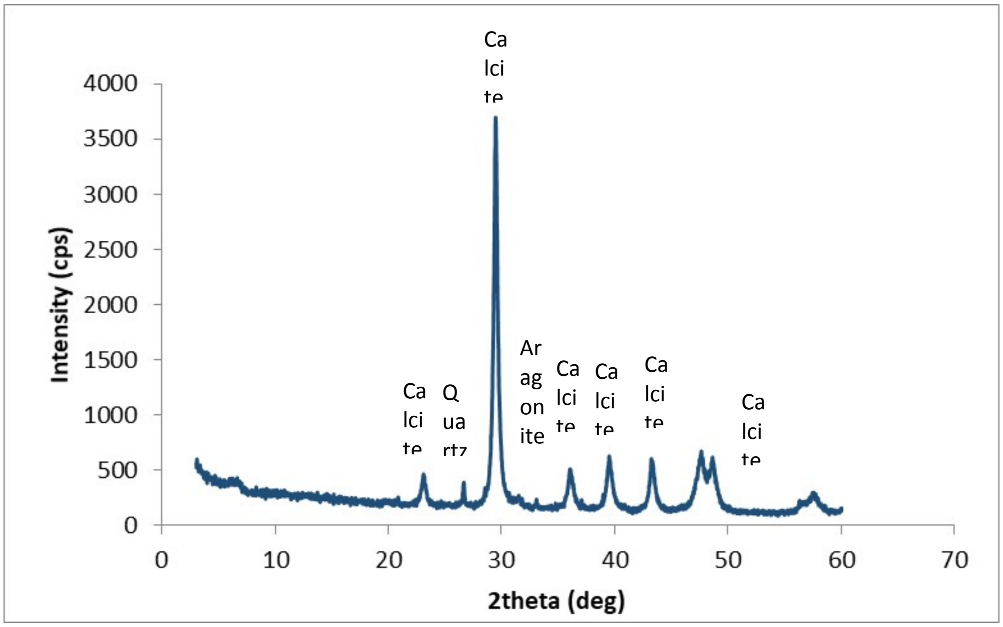

Fig. S2. XRD of carbonate rock

Table S2. Composition of Sea Water and its subsequent dilution

Ions	FW (mmol/l)	SW (mmol/l)	2dSW (mmol/l)	4dSW (mmol/l)	8dSW (mmol/l)	10dSW (mmol/l)	20dSW (mmol/l)	40dSW (mmol/l)
Na^+	446.37	459.37	229.685	114.843	57.421	45.937	22.969	11.484
Cl^-	488.08	535.82	267.91	133.955	66.978	53.582	26.791	13.396
Mg^{2+}	9.62	52.511	26.256	13.128	6.564	5.251	2.626	1.313
SO_4^{2-}	1.19	27.456	13.728	6.864	3.432	2.746	1.373	0.686
Ca^{2+}	11.77	9.911	4.956	2.478	1.239	0.991	0.496	0.248
K^+	0	8.8528	4.426	2.213	1.107	0.885	0.443	0.221
HCO^{3-}	7.40	2.38	1.19	0.595	0.298	0.238	0.119	0.0595
TDS (ppm)	28567	34360	17180	8690	4295	3436	1718	859
IS (mol/l)	0.5161	0.683	0.3415	0.1707	0.0859	0.0683	0.03415	0.017075
pH@25℃	6.65	7.65	7.61	7.48	7.39	7.28	6.93	6.60

Table S3 - Composition of ion tuned sea water

Components	20dSW* $2SO_4^{2-}$ (mmol/l)	20dSW* $3SO_4^{2-}$ (mmol/l)	20dSW* $4SO_4^{2-}$ (mmol/l)	20dSW* $2Ca^{2+}$ (mmol/l)	20dSW* $3Ca^{2+}$ (mmol/l)	20dSW* $4Ca^{2+}$ (mmol/l)	20dSW* $2Mg^{2+}$ (mmol/l)	20dSW* $3Mg^{2+}$ (mmol/l)	20dSW* $4Mg^{2+}$ (mmol/l)
Na^+	21.5392	20.1268	18.8679	20.6719	20.0194	18.5247	14.8623	7.1028	0.11882
Cl^-	22.2048	18.4619	14.4542	25.4963	25.8365	25.3330	24.3568	21.4261	19.6889
$Mg2^+$	**2.6249**	**2.6249**	**2.6249**	**2.6249**	**2.6249**	**2.6249**	5.2499	7.8749	10.4998
SO_4^{2-}	2.7462	4.193	5.4924	**1.3731**	**1.3731**	**1.3731**	**1.3731**	**1.3731**	**1.3731**
Ca^{2+}	**0.4965**	**0.4965**	**0.4965**	0.9930	1.4895	1.9861	**0.4965**	**0.4965**	**0.4965**
K^+	0.4425	0.4425	0.4425	0.4425	0.4425	0.4425	0.4425	0.4425	0.4425
HCO^{3-}	0.1188	0.1188	0.1188	0.1188	0.1188	0.1188	0.1188	0.1188	0.1188
TDS (ppm)	1655	1621	1582	1639	1656	1623	1509	1290	1132
IS (mol/l)	0.03423	0.03423	0.03423	0.03423	0.03423	0.03423	0.03423	0.03423	0.03423

Table S4 - Petrophysical properties of cores

Core Number	Length (cm)	Dia (cm)	Porosity (vol %)	Brine Permeability (md)	Connate Water Saturation (%)
1.	7.5	3.84	16.20	1.32	25.30
2.	7.5	3.84	15.83	1.87	18.69
3.	7.5	3.84	16.80	1.55	20.03
4.	7.5	3.84	16.43	1.52	24.83
5.	7.5	3.84	16.01	1.48	21.46

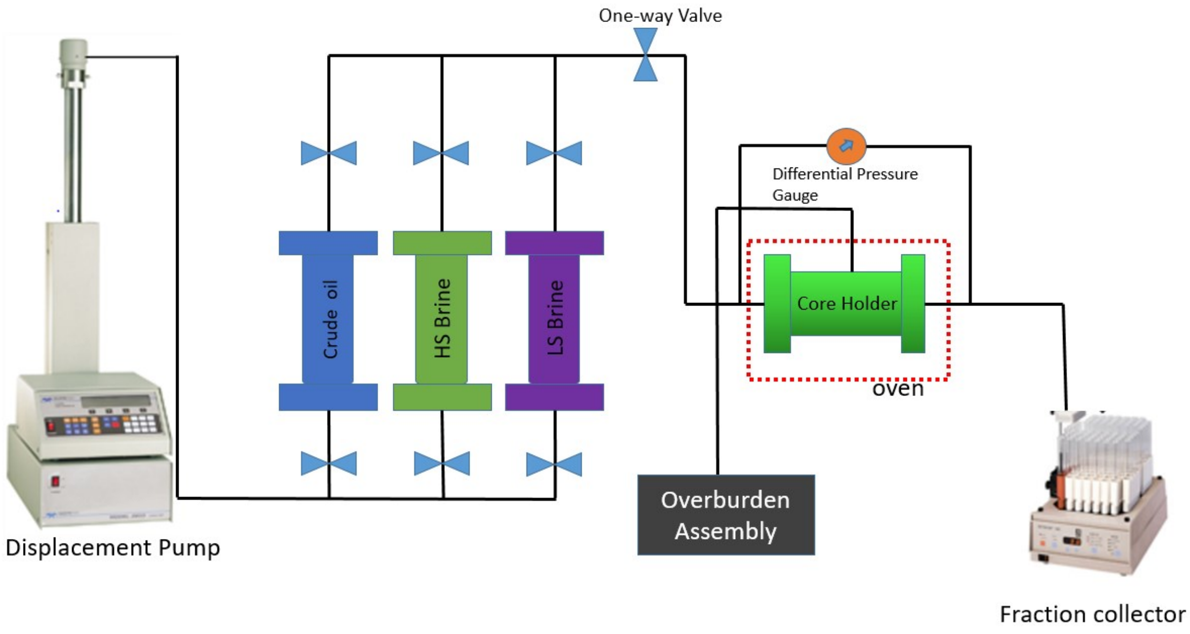

Fig. S3. Core Flooding Setup

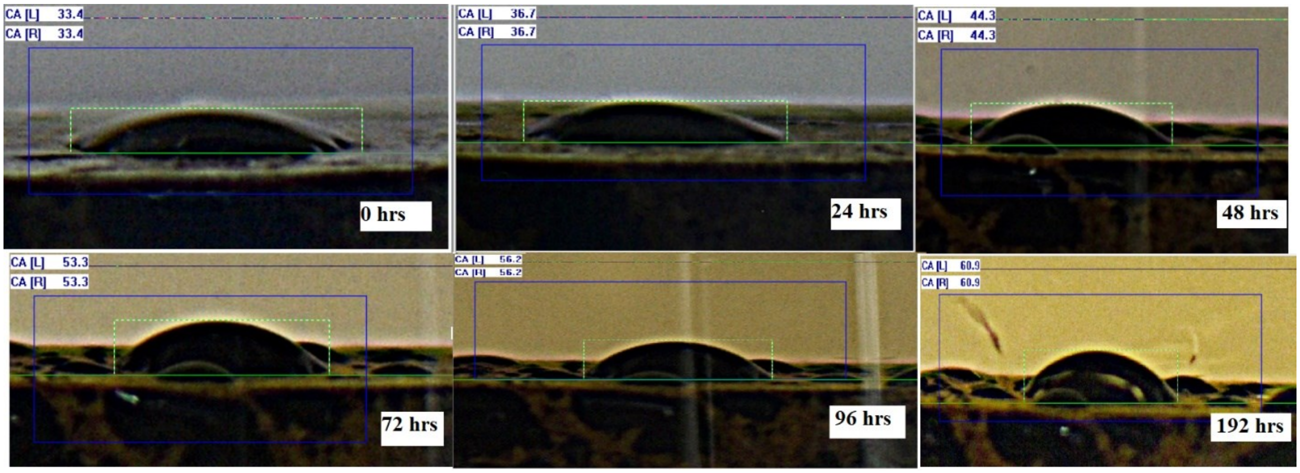

Fig. S4. Time variant contact angle of 20dSW (optimum salinity) at 90℃
